# Trends in Obesity Among Canadian Adults: A Population-Level Database Analysis

**DOI:** 10.7759/cureus.90206

**Published:** 2025-08-16

**Authors:** Violet C Mokwenye, Judith N Ochuba, Oluwatomiwa S Fasoro, Amarachi S Nwokolo, Oghale Osula, Marvellous O Sowunmi, Chidimma I. Oramu, Sharokeen Youkhana, Kelechukwu P Oranu, Amarachi Asomugha-Okwara, Okelue E Okobi

**Affiliations:** 1 Department of General Practice, University of Uyo, Uyo, NGA; 2 Department of Psychiatry, Internal Medicine, Surgery, Obstetrics, and Gynecology, National Hospital Abuja, Abuja, NGA; 3 Department of Internal Medicine, University of Benin Teaching Hospital, Benin City, NGA; 4 Department of Clinical Sciences, College of Health Sciences, Obafemi Awolowo University, Ifẹ, NGA; 5 Department of General Practice, Madonna University, Elele, NGA; 6 Department of General Medicine, University of Benin, Benin City, NGA; 7 Faculty of Medicine, Kharkiv National Medical University, Kharkiv, UKR; 8 Department of Psychiatry and Behavioral Sciences, Priory Hospital Mildmay Oaks, Hampshire, GBR; 9 Department of General Medicine, Hawler Medical University, Erbil, IRQ; 10 Department of Obstetrics and Gynecology, Kenechukwu Specialist Hospital and Maternity Enugu, Enugu, NGA; 11 School of Epidemiology and Public Health, University of Ottawa, Ottawa, CAN; 12 Department of Family Medicine, Deer Ridge Family Clinic, Calgary, CAN; 13 Department of Family Medicine, Larkin Community Hospital Palm Springs Campus, Miami, USA; 14 Department of Family Medicine, IMG Research Academy and Consulting LLC, Homestead, USA

**Keywords:** age and sex disparities, bmi, canada, chms, epidemiology, obesity, oecd, public health

## Abstract

Background and objective

Obesity continues to pose a critical public health challenge in Canada, contributing to the growing burden of chronic diseases such as type 2 diabetes, cardiovascular disorders, and mental health conditions. It is associated with reduced quality of life and increased healthcare expenditures. Despite awareness and policy efforts, national prevalence rates have remained elevated over the past decade. This study aims to assess 12-year trends in adult obesity in Canada from 2007 to 2019 and examine differences in prevalence by sex and age groups using national survey data and BMI-based classifications. A secondary objective was to benchmark Canada’s adult obesity prevalence against Organisation for Economic Co-operation and Development (OECD) countries (using the most recent national estimates available through 2023) and to identify how Canada compares with peer nations.

Methods

A cross-sectional, population-level analysis was conducted using data from Statistics Canada’s Canadian Health Measures Survey, cycles 1-6 (2007-2019), and complementary Statistics Canada reports. Adults aged 18 to 79 years were included. Descriptive statistics and 95% CIs were used to analyze prevalence trends over time and between demographic groups. OECD data from 2023 and earlier were used for international benchmarking.

Results

In 2019, the overall obesity prevalence among Canadian adults was 24.3% (95% CI: 18.0-32.0). Males had a higher prevalence (26.7%) than females (22.0%). Obesity increased with age: 19.8% in ages 18-39, 25.5% in 40-59, and 29.3% in 60-79. From 2007 to 2019, obesity varied within a narrow range, peaking in 2014-2015 (25.9%) before declining slightly. Compared internationally, Canada’s rate was similar to the OECD average (25%) but lower than countries like the US (42.8%).

Conclusions

Obesity remains a prevalent and complex issue in Canada, with significant variation across sex and age groups. Canada’s 2019 prevalence (24.3%) is close to the OECD average but contrasts with both higher-prevalence countries (e.g., the US) and lower-prevalence countries (e.g., several East Asian nations), underscoring the need for integrated clinical care and policy action tailored to national and subpopulation needs.

## Introduction

Obesity is a complex, chronic health condition characterized by excessive or abnormal fat accumulation that presents a risk to health [1]. In recent decades, obesity has emerged as a major public health concern worldwide, including in high-income countries like Canada [2]. With its increasing prevalence and multifaceted impact on health systems, economies, and individual well-being, obesity is now considered one of the leading drivers of preventable morbidity and mortality [3]. It is not merely a cosmetic concern but a disease entity that requires long-term management and integrated intervention strategies [4]. In Canada, nearly one in four adults aged 18 to 79 years is living with obesity, underscoring the urgent need to better understand the evolving trends and determinants of this condition to shape effective prevention and treatment policies [5].

In 2019, high BMI contributed to five million noncommunicable disease-related deaths. Globally, obesity rates have surged: between 1990 and 2022, obesity among children and adolescents (ages 5-19) rose from 2% to 8%, while adult obesity more than doubled from 7% to 16% [2]. According to the 2017-2020 US report, 42.4% of adults and 20.9% of youth have obesity. Additionally, 9.2% of adults have severe obesity (BMI ≥ 40 kg/m²) [6]. In Canada, adult obesity has tripled since 1985. By 2016, 26.4% (8.3 million) of adults were obese (BMI ≥ 30), and severe obesity (BMI ≥ 35) had risen by 455%, affecting 1.9 million. Additionally, 34% (10.6 million) of adults were overweight (BMI 25-29.9) [7].

At its core, obesity is a state of chronic positive energy balance, where caloric intake consistently exceeds expenditure. However, its development and persistence involve more than just lifestyle choices [8]. Pathophysiologically, obesity is associated with hormonal imbalances, including elevated leptin and insulin resistance (IR), along with dysregulated adipokine production, such as increased TNF-α and IL-6 from hypertrophic adipose tissue [9]. These changes trigger systemic inflammation and contribute to metabolic dysregulation [10]. At the molecular level, mitochondrial dysfunction in adipocytes, oxidative stress, and epigenetic modifications play central roles in the progression of obesity to related comorbidities like type 2 diabetes, hypertension, cardiovascular disease, non-alcoholic fatty liver disease, and certain cancers [11]. Moreover, obesity impairs hypothalamic signaling responsible for appetite and satiety regulation, creating a self-perpetuating cycle of increased hunger and decreased energy expenditure. From a clinical standpoint, these alterations highlight why treating obesity is not simply a matter of willpower but a medically significant condition requiring targeted, long-term interventions [12].

This paper has three interrelated objectives. First, to analyze temporal trends in adult obesity among Canadians aged 18 to 79 years between 2007 and 2019 (Canadian Health Measures Survey (CHMS) cycles 1-6), using measured BMI classifications to identify sustained increases, decreases, or stabilization in prevalence. Second, to quantify disparities in obesity prevalence by sex and by age groups (18-39, 40-59, and 60-79) in 2019, using 95% CIs to assess statistical differences. Third, to benchmark Canada’s adult obesity prevalence against Organisation for Economic Co-operation and Development (OECD) countries using the most recent national estimates available through 2023, with the goal of positioning Canada relative to peer nations for policy-relevant interpretation and comparative learning.

In addition to statistical analysis, the discussion section will contextualize these findings with clinical implications, case studies, and references to Canadian and US guidelines such as those by Obesity Canada, the Canadian Task Force on Preventive Health Care, and the American Association of Clinical Endocrinology (AACE). Ultimately, the study seeks to underscore obesity as a medical, social, and policy issue that requires coordinated, multidisciplinary action.

## Materials and methods

Data source and study design

This study utilized nationally representative data from the CHMS and Statistics Canada’s health indicator reports, which track adult obesity trends using BMI classifications [13]. BMI is calculated by dividing an individual’s weight in kilograms by the square of their height in meters (kg/m²), with values ≥30 indicating obesity [13]. The CHMS is a nationally representative cross-sectional survey designed to assess the health and wellness of Canadians through a combination of interviews, physical measurements, and laboratory testing. This analysis focused specifically on data collected between 2007 and 2019, encompassing six cycles of data collection. The study employed a descriptive, population-based design to examine national obesity trends over time and assess prevalence variations by sex and age in 2019. The study adhered to the standard definitions of obesity set by WHO and Canadian guidelines, using BMI thresholds to classify weight status.

Study participants and questionnaires

Study participants included Canadian adults aged 18 to 79 years, sampled across the various cycles of the CHMS. The survey uses a stratified multistage sampling design to ensure representation of the Canadian population, excluding those living in institutions, on reserves, or in remote regions. Participants were invited to complete structured health interviews and undergo physical assessments at mobile examination centers. Questionnaires captured demographic data (e.g., age and sex), lifestyle factors (e.g., physical activity, smoking, and diet), and self-reported health conditions. For this study, we limited our analysis to respondents with complete anthropometric data necessary for BMI calculation.

Data collection and quality assurance

Anthropometric measurements were taken by trained health professionals following standardized procedures. Height and weight were measured directly using calibrated stadiometers and digital scales. BMI was then calculated using the formula: weight (kg) divided by height (m²). Data integrity was maintained through regular calibration of instruments, double-checking of entries, and consistency checks across survey cycles. Statistics Canada applies sampling weights to ensure that the data represent the national population, accounting for non-response and complex survey design. Quality assurance processes also included verification protocols and post-collection adjustments to reduce bias and improve precision.

Variables of interest

The primary variable of interest was the prevalence of obesity, defined as a BMI of ≥30 kg/m², following WHO and national clinical standards. Secondary variables included age groupings (18-39, 40-59, and 60-79 years) and sex (male and female). For temporal trend analysis, data were grouped into six two- or three-year intervals (e.g., 2007-2009, 2009-2011, etc.) to align with survey cycle reporting. CIs (95% CI) were used to capture the statistical uncertainty around each prevalence estimate.

Data analysis and statistical methods

Descriptive statistics were used to summarize obesity prevalence over time and across demographic subgroups. Cycle-specific, survey-weighted point estimates and 95% CIs were computed using CHMS sampling weights to produce nationally representative estimates. For descriptive comparisons, we reported non-overlapping 95% CIs as an initial indicator of potential differences between groups.

To formally assess temporal trends, we used survey-weighted logistic regression models that accounted for the CHMS complex sampling design (strata and primary sampling units) and applied the provided sampling weights. Obesity status (BMI ≥ 30 kg/m²) was modeled as the binary outcome, and survey cycle (entered as a continuous variable representing the mid-year of each cycle) was the primary predictor to test for a linear time trend. We report adjusted ORs per one-year increase in cycle mid-year, 95% CIs, and Wald test p-values. To evaluate possible non-linear trends, we additionally fitted models including a quadratic term for cycle year and compared model fit using design-based Wald tests. Pairwise comparisons across cycles (e.g., peak vs. baseline) were performed using adjusted Wald tests when needed.

Subgroup trend analyses stratified by sex and by age group were conducted using the same survey-weighted logistic regression framework; we also tested interaction terms between cycle year and subgroup indicators to determine whether temporal trends differed significantly by sex or age. Age-standardized prevalence estimates (direct standardization) were produced using the 2011 Canadian population as the reference to facilitate comparisons across cycles with differing age distributions.

All statistical tests were two-sided with significance set at α = 0.05. Analyses were performed using IBM SPSS Statistics for Windows, Version 30.0 (Released 2024; IBM Corp., Armonk, NY, USA) for descriptive tabulations and Stata/SE 17 (StataCorp LLC, College Station, TX, USA; survey commands) for design-based regression modeling and formal hypothesis testing. Data visualization (trend figures) used Stata (StataCorp LLC) and Microsoft Excel (Microsoft Corporation, Redmond, WA, USA).

Ethical considerations

As this study involved secondary analysis of publicly available, de-identified data, no additional ethical approval was required. All CHMS protocols had been reviewed and approved by Health Canada and the Public Health Agency of Canada Research Ethics Board. The study complies with the Tri-Council Policy Statement (TCPS2) on ethical conduct for research involving humans. Confidentiality, privacy, and responsible use of data were maintained throughout the analysis.

## Results

The results reveal significant variations in adult obesity prevalence in Canada based on sex, age, and time, with Canada ranking near the OECD average and showing age-related and gender disparities. All group comparisons and trend tests reported below are based on survey-weighted, design-based tests that account for CHMS strata, clusters, and sampling weights.

Overall obesity prevalence in Canada (2019)

In 2019, the overall prevalence of obesity among Canadian adults aged 18 to 79 years was 24.3%, with a 95% CI (95% CI: 18.0-32.0). This indicates that nearly one in four Canadian adults was living with obesity during this period. The range between the lower and upper confidence limits reflects variability in population estimates, which may be influenced by sampling differences across regions and subgroups.

Prevalence of obesity based on gender (2019)

**Figure 1:**
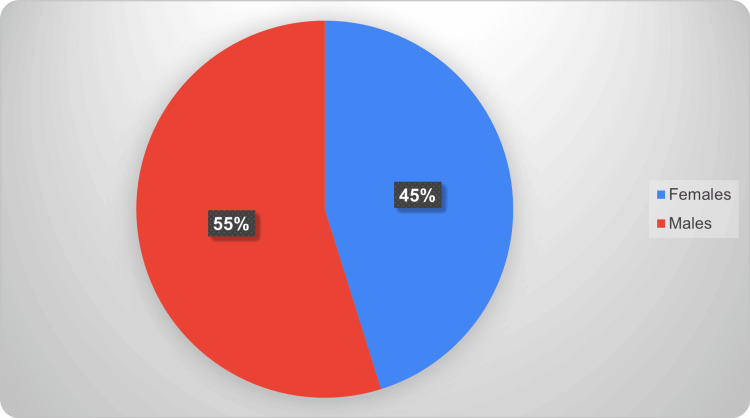
Gender distribution of obesity among Canadian adults (2019)

In 2019, males had a higher measured obesity prevalence (26.7%, 95% CI: 19.7-35.0) than females (22.0%, 95% CI: 15.0-30.9). A design-based Wald chi-square test comparing prevalence by sex indicated that this difference was statistically significant (survey-weighted Wald test, p < 0.05). To further quantify the difference, a survey-weighted logistic regression model (obesity status as outcome and sex as predictor) produced a statistically significant association between male sex and higher odds of obesity in 2019 after accounting for the CHMS design (p < 0.05). Figure 1 shows the prevalence of obesity by gender among Canadian adults (18-79 years) in 2019, showing higher rates in males than females with 95% CIs.

Prevalence of obesity by age group (2019)

The prevalence of obesity in Canada in 2019 also varied significantly by age group, showing a clear upward trend with increasing age. Among adults aged 18 to 39 years, the obesity rate was 19.8% (95% CI: 27.7-13.8), the lowest among all age groups. The relatively wide CI suggests variability in prevalence within this younger population, possibly due to lifestyle diversity, regional disparities, or early-stage onset of weight gain.

**Figure 2:**
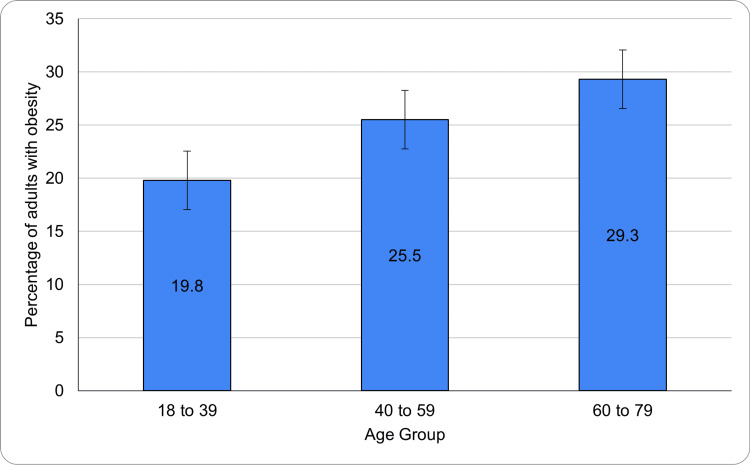
Age-wise distribution of obesity among Canadian adults (2019)

In the middle-aged group of 40 to 59 years, obesity prevalence increased to 25.5% (95% CI: 36.5-16.9). This group demonstrated the broadest CI, indicating a wide spread in data and potentially capturing individuals transitioning between lifestyle stages and health status. Finally, the 60 to 79 years group exhibited the highest obesity prevalence at 29.3% (95% CI: 40.7-20.0), suggesting that older adults face the greatest burden of obesity-related health risks. This progression aligns with the physiological slowing of metabolism, decreased physical activity levels, and age-related hormonal changes that influence body composition. Figure 2 shows the prevalence of obesity across different age groups (18-39, 40-59, 60-79) among Canadian adults in 2019. Obesity rates increased with age, with the highest prevalence observed in the 60-79 age group.

Trends over time (2007-2019)

**Figure 3:**
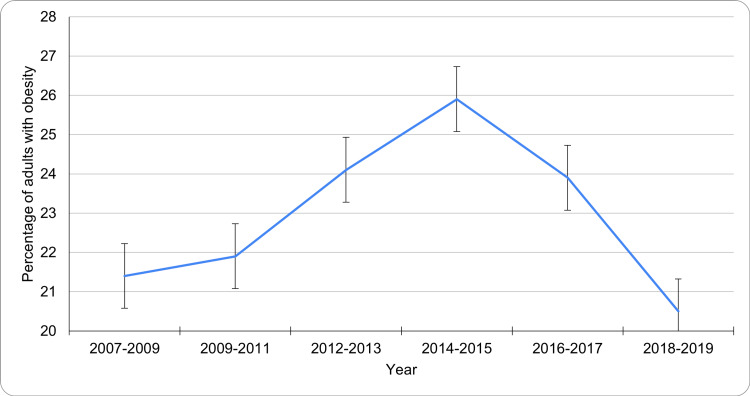
Trends in adult obesity prevalence in Canada (2007-2019)

Longitudinal data from 2007 to 2019 show varying patterns in obesity prevalence among Canadian adults. The decade began with a prevalence of 21.4% during 2007-2009 (95% CI: 23.6-19.2), which gradually rose to 21.9% in 2009-2011 (95% CI: 25.2-18.6), and then climbed to 24.1% in 2012-2013 (95% CI: 28.0-20.2). The peak was observed in 2014-2015, reaching 25.9% (95% CI: 29.7-22.1), after which there was a gradual decline to 23.9% in 2016-2017 (95% CI: 27.2-20.6) and 20.5% in 2018-2019 (95% CI: 24.1-16.9). Figure 3 shows the trend in obesity prevalence among Canadian adults aged 18-79 from 2007 to 2019. While rates fluctuated slightly, the overall prevalence remained high throughout the period.

When examining the trend in obesity over the decade prior, Canada's adult obesity rate showed fluctuations. From 2007 to 2009, the prevalence stood at 21.4% (95% CI: 23.6-19.2). This figure gradually rose to 25.9% by 2014-2015 (95% CI: 29.7-22.1), marking the peak within this observation window. A slight decline followed in 2016-2017, where the prevalence fell to 23.9% (95% CI: 27.2-20.6), and by 2018-2019, the rate dropped further to 20.5% (95% CI: 24.1-16.9). These trends indicate that although there was a rise in obesity prevalence during the first half of the decade, efforts in public health policy and awareness may have contributed to a modest decrease in more recent years. Nevertheless, nearly one in four adults in Canada was living with obesity in 2019, underscoring a significant public health challenge.

International comparison (2023 or latest year available)

In a global context, Canada’s obesity prevalence of 24.3% in 2019 is broadly comparable to the OECD average of 25%, placing the country in the mid-tier among developed nations [13]. In contrast, countries like the United States (42.8%, 2018) and Mexico (36%, 2020) reported significantly higher obesity rates, categorizing them as worse-performing nations. Similarly high rates were observed in Chile (34.4%, 2016) and Hungary (33.2%, 2019). Table 1 presents the prevalence of adult obesity (BMI ≥ 30 kg/m²) across OECD countries, organized in ascending order by year of data collection (2010-2023). The data include the most recently available national estimates for each country, highlighting variation in obesity rates, with the highest reported in the United States (42.8%) and the lowest in Japan (4.6%). The OECD average stands at 25.0%.

**Table 1:** Prevalence of obesity among adults (aged 15 and older) in OECD countries by year of latest available data -: not available

Year	Country	Obesity prevalence (%)
2010	Czech Republic	21
2012	Germany	23.6
2014	Estonia	18
2015	Portugal	28.7
2015	Israel	18.8
2015	Colombia	18.7
2016	Chile	34.4
2017	Türkiye	28.8
2017	France	15.6
2018	United States	42.8
2018	Costa Rica	31.2
2018	United Kingdom	28
2018	Belgium	21.2
2019	Hungary	33.2
2019	Canada	24.3
2019	Ireland	23
2019	Japan	4.6
2020	Mexico	36
2022	Australia	30.7
2022	Finland	30.2
2022	Latvia	23.3
2022	Korea	7.1
2023	New Zealand	32.6
-	OECD average	25

Canada’s obesity prevalence (24.3%) is lower than that of countries like New Zealand (32.6%, 2023), Costa Rica (31.2%, 2018), Australia (30.7%, 2022), and the United Kingdom (28%, 2018). Slightly lower figures were seen in Germany (23.6%, 2012) and Ireland (23%, 2019). Meanwhile, countries such as France (15.6%, 2017), Estonia (18%, 2014), Japan (4.6%, 2019), and Korea (7.1%, 2022) demonstrated significantly better outcomes. The contrast is most striking with East Asian nations, where cultural dietary patterns, high physical activity, and lower processed food consumption contribute to substantially lower obesity rates.

This comparison places Canada in a moderate-risk category, necessitating sustained policy-level actions, including food environment regulations, community-level health promotion, and obesity management programs.

## Discussion

This discussion explores the findings of a population-level trend analysis on adult obesity in Canada, emphasizing demographic patterns, temporal variations, clinical implications, molecular mechanisms, and public health responses. With 24.3% of Canadian adults living with obesity in 2019, the condition remains a critical public health concern. By synthesizing epidemiological data with clinical case examples, guideline comparisons, and mechanistic insights, this section underscores obesity as a multifactorial disease requiring patient-centered care and structural interventions. Additionally, this study positions Canadian trends within international benchmarks, identifying actionable areas for health system improvement.

Building on the national trend observed, demographics such as age and sex emerged as strong predictors of obesity in Canada. The study demonstrated that obesity prevalence increased progressively with age, ranging from 19.8% in the 18-39 age group to 25.5% in those aged 40-59 and peaking at 29.3% among those aged 60-79. These between-group differences were evaluated using design-based Wald chi-square tests and survey-weighted logistic regression models that accounted for the CHMS complex sampling design, and both age group and sex were statistically associated with obesity status in 2019 (p < 0.05). Because our models did not adjust for socioeconomic status, ethnicity, or other potential confounders, we present these results as cross-sectional associations rather than as causal predictors. These trends align with global research showing that advancing age correlates with decreased basal metabolic rate, reduced lean muscle mass, and cumulative exposure to obesogenic environments [14]. Men exhibited higher obesity prevalence (26.7%) compared to women (22.0%), a pattern corroborated by national surveillance data and international findings [15]. Biological factors, including hormonal influences such as testosterone decline in men, as well as behavioral and occupational differences, may account for this disparity [16,17].

While individual behaviors and biological factors explain part of the disparity, social determinants of health provide a deeper context for the population-level variations in obesity. Populations with limited income or educational attainment are more likely to reside in neighborhoods with poor access to nutritious food, safe physical activity spaces, and healthcare services. Food deserts, housing instability, and employment in sedentary or shift-based jobs exacerbate risk [18]. Indigenous peoples, recent immigrants, and racialized minorities face additional challenges due to systemic inequities, historical trauma, and cultural disconnects in healthcare delivery [19,20]. These disparities underscore the importance of equity-focused strategies in obesity prevention and treatment.

In addition to demographic disparities, examining the temporal evolution of obesity prevalence reveals noteworthy trends over the past decade. Temporal analysis from 2007 to 2019 indicates that obesity prevalence in Canadian adults remained relatively stable, with a peak of 25.9% in 2014-2015 and a subsequent decline to 20.5% by 2018-2019. This plateauing trend mirrors findings in other high-income countries such as the United States and the United Kingdom [21]. The modest reduction observed may reflect increasing awareness and implementation of policy interventions, such as improved nutrition labeling, public discourse around sugar taxation, and community efforts promoting physical activity [22]. Still, the persistently high rates indicate that education and awareness alone are insufficient for meaningful population-level change [23].

Placing Canada’s obesity trends in a global context highlights both shared challenges and unique national circumstances. Canada’s 24.3% obesity prevalence is comparable to the OECD average of 25%, yet remains lower than the United States (42.8%) [24]. Differences in healthcare systems, social support programs, and food policies may contribute to these cross-national variations. While Canada’s universal healthcare system and stronger social safety nets may mitigate some obesity risk factors, the overall burden remains substantial, warranting comprehensive national strategies.

Regardless of geography, the clinical burden of obesity remains profound, extending beyond BMI classification to include a wide range of metabolic, cardiovascular, musculoskeletal, and psychological consequences. At the population level, the clustering of comorbidities such as type 2 diabetes, hypertension, sleep apnea, and osteoarthritis is well documented among adults with obesity in Canada and internationally. National surveillance data and large-scale clinical trials, including SCALE and LEADER, provide evidence that comprehensive interventions, combining dietary modification, increased physical activity, behavioral therapy, and, where appropriate, pharmacologic agents such as GLP-1 receptor agonists, can produce meaningful improvements in weight, metabolic health, and quality of life [25,26]. These findings reinforce the need for public health and health system approaches that integrate preventive strategies with accessible, multidisciplinary treatment, moving from a solely weight-centric paradigm toward one that prioritizes functional capacity and quality-of-life outcomes across the population.

Although often portrayed as a result of poor lifestyle choices, obesity is underpinned by complex molecular and physiological mechanisms. White adipose tissue, particularly visceral fat, acts as an endocrine organ that undergoes hypertrophy and hyperplasia in obesity. This expansion leads to local hypoxia, macrophage infiltration, and fibrosis. Subsequently, adipose tissue secretes pro-inflammatory cytokines such as TNF-α, IL-6, and resistin, while reducing protective adipokines like adiponectin, contributing to IR, endothelial dysfunction, and systemic inflammation, a process termed metaflammation [27,28]. Adding to the hormonal complexities, obesity is associated with leptin resistance, where elevated leptin levels fail to suppress appetite due to impaired hypothalamic signaling [29]. Ghrelin dysregulation and central nervous system inflammation further exacerbate energy imbalance and weight gain. Mitochondrial dysfunction in adipocytes impairs fatty acid oxidation and increases reactive oxygen species production, contributing to cellular stress and metabolic impairment.

Recent research has highlighted the significant role of the gut microbiome in the pathogenesis of adult obesity, particularly through its influence on nutrient absorption, fat storage, metabolic regulation, and immune function. Dysbiosis, a reduction in microbial diversity and an imbalance between beneficial and pathogenic bacteria, has been linked to chronic low-grade inflammation, altered gut barrier function, IR, and increased fat accumulation in both mouse models and human studies [29-31]. In Canada, where obesity rates continue to climb, these findings open new avenues for intervention. Future strategies may include microbiome-targeted approaches such as personalized nutrition plans, probiotics or prebiotics, and continued research into microbiome-based therapies that account for the country’s diverse population and healthcare landscape.

Obesity care has evolved from simplistic advice to “eat less and move more” toward recognizing the chronic, relapsing nature of the condition. The 2020 Canadian Adult Obesity Clinical Practice Guidelines represent a paradigm shift in this regard. Developed by Obesity Canada and the Canadian Association of Bariatric Physicians and Surgeons, the guidelines emphasize personalized care through the 4Ms framework: Mental (psychological well-being), Mechanical (physical functioning), Metabolic (cardiometabolic health), and Milieu (social determinants). They advocate the use of the Edmonton Obesity Staging System (EOSS) to assess disease severity based on functional impairment rather than BMI alone [32].

This patient-centric approach includes individualized goal-setting, shared decision-making, and multidisciplinary intervention. Recommended therapies include dietary modification tailored to cultural preferences, structured physical activity plans, pharmacotherapy with agents like semaglutide or liraglutide, and bariatric surgery in select patients. Clinical outcomes are evaluated not solely through weight loss, but through improvements in glycemic control, blood pressure, functional mobility, and psychological health [32].

The AACE and The Obesity Society in the United States also emphasize obesity as a chronic disease. However, Canadian guidelines place comparatively greater weight on patient-reported outcomes, stigma reduction, and function over aesthetics or numeric targets [33].

While individualized care is essential, policy-level actions are equally critical to creating environments that support healthier choices. Taxes on sugar-sweetened beverages, mandatory front-of-package labeling, marketing restrictions on junk food, and subsidies for fruits and vegetables can create supportive environments for healthy choices [34,35]. Urban design that promotes walkability and physical activity, as well as food literacy campaigns in schools, can shift population behaviors.

To ensure these policies reach all Canadians equitably, strategies must also be inclusive and responsive to systemic barriers. Studies by Jackson et al. found that obesity prevalence is higher among Indigenous and low-income populations in Canada, often due to intergenerational trauma, systemic barriers, and under-resourced communities [20]. Policies must thus go beyond behavioral recommendations to address structural and social inequities.

Future research should adopt a longitudinal perspective to track obesity onset, progression, and response to interventions over time. Integrating advanced diagnostics such as dual-energy X-ray absorptiometry, MRI-based body composition analysis, and inflammatory biomarkers will enhance clinical risk stratification and treatment monitoring [36].

Randomized controlled trials investigating next-generation anti-obesity medications (e.g., dual GLP-1/GIP receptor agonists like tirzepatide) are needed to expand therapeutic options. Moreover, research into the gut microbiome, genetic polymorphisms, and neuroendocrine signaling could unlock new pathways for intervention [25,26].

Evaluations of public health policies in real-world contexts, such as sugar taxes in Mexico or menu labeling in the UK, provide valuable insights into population-level effects. In Canada, rigorous outcome assessments of similar programs can inform future policymaking. Finally, inclusive research that includes marginalized populations will be essential to reduce health disparities and improve obesity outcomes for all Canadians [13].

Canadian clinical guidelines recommend pharmacological treatments for obesity, specifically GLP-1 receptor agonists such as liraglutide and semaglutide, in addition to behavioral and nutritional interventions for adults with a BMI of 30 kg/m² or ≥27 kg/m² with complications related to adiposity [37]. Although guidelines have been included and regulatory licenses have been granted (liraglutide in 2015, naltrexone-bupropion in 2018, and semaglutide for obesity in 2021), real-world uptake is still low: the reimbursement of public drug plans is severely limited between provinces, and less than 20% of Canadians with private insurance have coverage. According to Ontario Clinic data from 2015 to 2018, just about 25% of patients received psychological or surgical treatments [38].

IR affects key metabolic tissues such as the liver, muscle, and adipose tissue, leading to reduced glucose uptake and utilization. As a result, the pancreas compensates by increasing insulin secretion, which leads to hyperinsulinemia. Elevated insulin levels accelerate fat accumulation by promoting triglyceride synthesis, inhibiting fat breakdown, and supporting adipocyte differentiation [39]. At the same time, expanding visceral fat releases fatty acids and inflammatory cytokines that further impair insulin signaling in the liver and muscle, creating a vicious cycle where obesity and IR reinforce each other [40]. Aging contributes to IR through mechanisms such as nitric oxide synthase-mediated interference in insulin signaling. These pathways further compound obesity-related metabolic risks in older adults. This rise in iNOS promotes S-nitrosation of key proteins in the insulin signaling pathway, impairing their function and reducing insulin sensitivity. Studies in aged mice show that those lacking iNOS are protected from this effect, and interventions such as iNOS inhibition or exercise can restore insulin responsiveness by reducing S-nitrosation [41].

While IR affects both men and women, there are notable sex differences in its prevalence, causes, and consequences. IR is generally more common in men than in premenopausal women, likely due to the protective effects of estrogen on insulin sensitivity. However, after menopause, women experience a decline in estrogen levels, and their risk of IR increases to match that of men. Differences in body fat distribution also play a role: men typically accumulate more visceral fat, which is strongly associated with IR, whereas women usually store more subcutaneous fat, which is metabolically less harmful [41]. These mechanisms may help explain the higher prevalence of obesity in older adults and the age- and sex-related disparities observed in Canadian adult data.

Strengths and limitations 

This study provides a comprehensive population-level analysis of adult obesity trends in Canada using data from the CHMS and Statistics Canada. A notable strength is the use of nationally representative, objectively measured anthropometric data collected by trained health professionals using standardized protocols, which enhances both accuracy and generalizability. The analysis spans 12 years (2007-2019), allowing for the examination of temporal trends, and stratification by age, sex, and survey cycle supports a detailed understanding of demographic differences in obesity prevalence. Situating Canadian findings within an international context using recent OECD data offers valuable benchmarking for global public health comparisons.

Several limitations should be acknowledged. First, the study uses BMI as the sole indicator of obesity, which does not distinguish between fat and lean mass or account for fat distribution. Second, as a cross-sectional analysis, the study cannot establish causality or follow individual weight changes over time. Third, while the study describes observed differences by age and sex, these are based on descriptive analyses and should not be interpreted as causal associations. Fourth, the lack of ethnicity, socioeconomic status, and regional subgroup analyses limits exploration of important social determinants and health inequities. Additionally, some subgroup estimates have relatively wide CIs, reflecting sampling variability. Lastly, the CHMS excludes institutionalized populations and individuals living on reserves, which may underrepresent certain high-risk groups. Future research could address these gaps through longitudinal, multidimensional data collection and expanded demographic coverage.

## Conclusions

Obesity remains a significant and persistent public health challenge in Canada, affecting approximately one in four adults aged 18 to 79 years. This study highlights demographic disparities, with higher prevalence among men and older adults, and modest fluctuations over the last decade. Although Canada’s rates are close to the OECD average, the health, social, and economic burdens of obesity warrant urgent and sustained attention. Beyond lifestyle factors, obesity is a complex, chronic disease with deep biological, environmental, and social determinants. Effective management must combine clinical care, including pharmacological and surgical options, with preventive strategies targeting upstream factors such as food environments, income inequality, and health literacy. National guidelines from Obesity Canada and international counterparts call for integrated, stigma-free care that prioritizes patient-centered outcomes. Moving forward, a coordinated, evidence-informed, and equity-focused approach is essential to reduce obesity prevalence and mitigate its long-term health consequences across the Canadian population.
